# Luminance and attentional modulation of size perception in peripheral vision: Response to Bedell and Johnson

**DOI:** 10.1167/jov.26.10.23

**Published:** 2026-10-26

**Authors:** Tristan Jurkiewicz, Yaffa Yeshurun, Laure Pisella

**Affiliations:** 1 Centre de Recherche en Neurosciences de Lyon (CRNL), INSERM U1028, CNRS UMR5292, Université Claude Bernard Lyon 1, Bron, France; 2 Hospices Civils de Lyon, Service de Neurocognition et de Neuro-ophtalmologie, Bron, France; 3 School of Psychological Science, University of Haifa, Haifa, Israel

We thank Bedell and Johnson for their comment (Bedell & Johnson, 2026) on our recent study and for drawing attention to their earlier work (Bedell & Johnson, 1984) on the influence of peripheral target luminance on the size-eccentricity effect (SEE). However, artists have long observed the underestimation of peripheral stimuli relative to central ones (e.g., Piles 1708, cited and illustrated in Baldwin, Burleigh, Pepperell, & Ruta, 2016, figure 1). Using dedicated experiments, it has been found and systematically reproduced by many authors with various stimuli, whether pictorial (Pepperell, 2012; Baldwin, Burleigh, & Pepperell, 2014; Koenderink, van Doorn, Pinna, & Pepperell, 2016) or laboratory shapes and whether shapes were black on a white background (Baldwin et al., 2016, first experiment; Kirsh, Heitling, & Kunde, 2018; Kirsch, Pfister, & Kunde, 2020; Kirsch, Kitzmann, & Kunde, 2021; Jurkiewicz, Salemme, Froment, & Pisella, 2021; Laaboudi, Hillairet de Boisferon, & Paeye, 2025), white on a black background (Han, Tan, Zhuang, & Qian, 2022; Jurkiewicz, Foncelle, Yeshurun, & Pisella, 2026), or red on a black background (Baldwin et al., 2016, second and third experiments), hence with various luminance, contrast, and hue. Bedell and Johnson's (1984) findings that the retinal coding of size is modulated by the manipulation of peripheral target luminance, resulting in a modulation and even a reversal of the SEE, seem limited to very specific experimental conditions. We therefore think that this retinal effect is unrelated to our theoretical question about the effect of visual attention.

Critically, as Bedell and Johnson acknowledge in their letter (Bedell & Johnson, 2026), there is no reason to suspect that the attention-based effect on perceived size we found would depend on the specific luminance of the targets and background we used. That is why we did not report this target luminance information in our 2021 or 2026 studies. However, in both studies, the contrasts were always the highest, either white on black (in 2026) or vice versa (in 2021). We used white on black in the 2026 study to reduce the visual strain associated with prolonged viewing of a white background due to the significant increase in the number of trials required in studies involving attentional manipulation in healthy controls. Our statement that the stimuli were the same as those used in Jurkiewicz et al. (2021) referred to their geometrical shape characteristics rather than their contrast polarity. We appreciate the opportunity to clarify this point. Importantly, luminance and contrast polarity were not experimental variables in either study and were held constant within each experiment: The 2021 article compared patients versus controls or contrasted right versus left visual fields, and the 2026 article contrasted valid versus invalid attentional cueing conditions.

In both our articles, our aim was to study the role of cortical spatial attention maps in the representations of stimulus size by testing the effect of parietal damage (Jurkiewicz et al., 2021) and the effect of attentional cueing (Jurkiewicz et al., 2026) on the SEE. In the visual areas, central vision is overrepresented relative to peripheral vision, a phenomenon also known as cortical magnification. In macaques, within a dorsal visual area, zone V6 contains a representation of the visual field showing little or no central cortical magnification (Galletti, Fattori, Gamberini, & Kutz, 1999). Its proposed human homologue in parietal areas also displays a large representation of the visual periphery (Pitzalis et al., 2013). These observations have led us to propose that dorsal parietal regions may contribute to compensating for the spatial distortions associated with central cortical magnification (Vindras et al., 2016), such as the SEE. The greater underestimation observed in the affected visual field in patients with optic ataxia supported a contribution of the dorsal parietal cortex to compensate for the effect of cortical magnification on magnitude perception in peripheral vision (Jurkiewicz et al., 2021). These areas of the dorsal posterior parietal cortex are also known for their role in endogenous spatial attention. In our 2026 study (Jurkiewicz et al., 2026), we therefore recruited control subjects to perform a similar size estimation task in the context of a Posner cueing paradigm (Posner, 1980). We had trials preceded by either a valid or an invalid cue. We found a larger underestimation of size in peripheral vision in the absence of spatial attention (invalid trials). This suggested that the underestimation of size in patients with ataxia may result from impairment of endogenous attentional mechanisms caused by damage to the dorsal posterior parietal cortex (Striemer et al., 2007). This also strengthened the argument that parietal-based attention is involved in size perception by compensating for cortical magnification.

Bedell and Johnson also put forward the hypothesis that the effect of attention on perceived size may result, in part, from changes in the detectability of the stimulus. Indeed, attention can affect detectability. However, we do not think that this is a plausible mechanism in our study because we are presenting white stimuli on a black background, ensuring the highest possible contrast, and therefore the stimuli were easily detected regardless of attention condition.

 Finally, we would be cautious about directly comparing the magnitude of the luminance-dependent effects reported by Bedell and Johnson (1984) with the cueing effects observed in our Figure 3. The studies differ substantially in their stimuli, presentation conditions, eccentricities, psychophysical procedures, and experimental manipulations. Most importantly, Bedell and Johnson systematically manipulated target luminance, whereas our attentional effect was obtained while the physical stimulus remained unchanged. Similarities in effect magnitude therefore do not establish a common underlying mechanism.

We thank Bedell and Johnson for highlighting another determinant of size perception. Their observations reinforce the view that perceived size is likely determined by multiple interacting factors. Several retinal, subcortical, and cortical maps may jointly determine perceived size across the visual field.
